# Inhibition of glutamate decarboxylase (GAD) by ethyl ketopentenoate (EKP) induces treatment-resistant epileptic seizures in zebrafish

**DOI:** 10.1038/s41598-017-06294-w

**Published:** 2017-08-03

**Authors:** Yifan Zhang, Michiel Vanmeert, Aleksandra Siekierska, Annelii Ny, Jubi John, Geert Callewaert, Eveline Lescrinier, Wim Dehaen, Peter A. M. de Witte, Rafal M. Kaminski

**Affiliations:** 1 Laboratory for Molecular Biodiscovery, Department of Pharmaceutical and Pharmacological Sciences, KU Leuven, Leuven Belgium; 2 REGA institute for Medicinal Chemistry, Department of Pharmaceutical and Pharmacological Sciences, KU Leuven, Leuven Belgium; 3 Molecular Design and Synthesis, Department of Chemistry, KU Leuven, Leuven Belgium; 4 Department of Cellular and Molecular Medicine, KU Leuven, Leuven Belgium; 5 Neuroscience TA, UCB Biopharma, Braine-l’Alleud, Belgium; 6 Organic Chemistry Section, CSTD, CSIR-NIIST, Thiruvananthapuram-19, Kerala, India

## Abstract

Epilepsy is a chronic brain disorder characterized by recurrent seizures due to abnormal, excessive and synchronous neuronal activities in the brain. It affects approximately 65 million people worldwide, one third of which are still estimated to suffer from refractory seizures. Glutamic acid decarboxylase (GAD) that converts glutamate into GABA is a key enzyme in the dynamic regulation of neural network excitability. Importantly, clinical evidence shows that lowered GAD activity is associated with several forms of epilepsy which are often treatment resistant. In the present study, we synthetized and explored the possibility of using ethyl ketopentenoate (EKP), a lipid-permeable GAD-inhibitor, to induce refractory seizures in zebrafish larvae. Our results demonstrate that EKP evoked robust convulsive locomotor activities, excessive epileptiform discharges and upregulated *c-fos* expression in zebrafish. Moreover, transgenic animals in which neuronal cells express apoaequorin, a Ca^2+^-sensitive bioluminescent photoprotein, displayed large luminescence signals indicating strong EKP-induced neuronal activation. Molecular docking data indicated that this proconvulsant activity resulted from the direct inhibition of both gad67 and gad65. Limited protective efficacy of tested anti-seizure drugs (ASDs) demonstrated a high level of treatment resistance of EKP-induced seizures. We conclude that the EKP zebrafish model can serve as a high-throughput platform for novel ASDs discovery.

## Introduction

Epilepsies are complex neurological disorders characterized by recurrent, unprovoked seizures resulting from the imbalance between excitatory and inhibitory neuronal processes occurring in the brain^1^. Despite the current therapeutic regimens and the availability of more than 20 anti-seizure drugs (ASDs), seizures are still poorly controlled in a significant proportion of patients (ca. 20–30%)^2^. Hence, there is a need for the generation of new *in vivo* models with the appropriate pathological background, to speed up the discovery of novel hits active against drug-resistant epilepsies^3, 4^.

Although traditional gatekeeper models like the maximal electroshock seizure and subcutaneous (s.c.) pentylenetetrazole (PTZ) seizure test have been instrumental for preclinical discovery of ASDs, these models may fail to identify novel compounds with improved efficacy against drug-resistant epilepsies^4^. ILEA defines drug resistant epilepsy as failure of two adequate trials of tolerated and appropriately chosen ASDs^5^. Consequently, an animal model can be considered as “drug resistant” if seizures do not respond (or respond poorly) to treatment with at least two current ASDs at maximum tolerated doses^4^. A number of promising rodent models which fulfill this definition have been developed over recent years^6^. Such models can be broadly categorized as those that rely on selection of ASD non-responders or those in which animals exhibit a poor drug response and in such case the models are “*per se*” resistant to ASDs^3, 4^. While some of these models are tunable to medium-throughput drug screens (e.g. the 6 Hz (44 mA) acute seizure model), most models (i.e. selection of non-responder animals in kindling or post status epilepticus models) are however difficult to implement because of their relatively high costs and labor-intensive procedures.

Zebrafish have received a great deal of attention over the last decade as a cost-efficient and relevant alternative for human disease modelling and large-scale drug screenings^7–9^. Besides ease of handling and fast reproduction rate, zebrafish share high genetic, cellular and organ homologies to humans^10^. Consequently, over recent years several chemical and genetic zebrafish models of acute seizures or epilepsy have been generated either by immersion of larvae in chemical proconvulsants like PTZ^11, 12^ or D-AG^13^, or by knocking down or introducing mutations in epilepsy susceptible genes including *lgi1* ^14^, *scn1Lab* ^15, 16^, and *kcnq3* ^17^.

Gamma-aminobutyric acid (GABA), the primary inhibitory neurotransmitter in the central nervous system (CNS), counterbalances neuronal excitability and hence plays a vital role in the predisposition/treatment of epilepsy^18^. Consequently, a substantial number of ASDs target the GABAergic system to suppress seizures through enhancing GABA-mediated inhibition (benzodiazepines, and barbiturates), inhibiting GABA catabolism (valproate and vigabatrin) or preventing GABA reuptake (tiagabine). Conversely, chemicals like PTZ, bicuculline, penicillin and picrotoxin that antagonize the GABAergic pathway can cause seizures^13, 19^.

GABA is bio-synthesized through oxidative decarboxylation of glutamate (Glu) catalyzed by the rate-limiting enzyme glutamate decarboxylase (GAD, EC 4.1.1.15). In mammals, GAD exists in two isoforms, GAD67 and GAD65^18, 20^ encoded by the *GAD1* and *GAD2* genes, respectively. Although both isoforms are co-expressed in GABAergic neurons, their expression levels and subcellular localization differ, and hence they likely play different roles in GABA-mediated neurotransmission^21^. Gad65^−/−^ mice are spared from major morphological defects and display no significant changes in brain GABA content, but these animals develop spontaneous seizures associated with increased mortality^22^ and show enhanced seizure susceptibility to picrotoxin and PTZ^23^. Gad67^−/−^ mice, on the other hand, show a 93% reduction in GABA concentration in the cerebral cortex. As these animals exhibit severe cleft palate resulting in neonatal death, their epileptic phenotype remains largely unknown^24^. *gad1b* and *gad2* are the zebrafish orthologues of human *GAD1* and *GAD2* and share around 76% homology. The encoded proteins gad67 and gad65 have been identified in brain and spinal cord tissue of developing zebrafish embryos and mediate local GABA synthesis^25, 26^. The zebrafish genome also includes a *gad3* gene that is considered as an ancient paralog of *GAD1* and *GAD2* that is lost in the hominid and rodent lineages. However, no specific role of *gad3* could be identified^27^.

Clinical evidence further shows that lowered GAD activity is associated with several forms of epilepsy. Reduced GAD activity has been found in epileptic foci from patients with intractable epilepsy, indicating that failure to synthesize GABA and loss of inhibitory synaptic activity may lead to epilepsy^28^. Furthermore, in autoimmune epilepsies, GAD antibodies have been detected especially in patients with focal epilepsies like drug-resistant temporal lobe epilepsy (TLE)^29–34^.

These data underscore the validity of decreasing GAD activity in zebrafish as an epilepsy-relevant paradigm for drug discovery. (D, L)-allylglycine (AG, 2-amino-4-pentenoic acid) is a GAD inhibitor that induces epileptic convulsions and neuronal damage in rodents and goldfish, and decreases the threshold for photic-induced seizures in baboons with photosensitive epilepsy^35–37^.

Recently, Leclercq *et al*. compared AG-induced acute seizures in zebrafish larvae and adult mice. The results showed clear cross-species similarities with regard to seizure behavior and demonstrated limited efficacy of ASDs^13^. A drawback of the AG zebrafish model is that seizures occurred asynchronously and with a long latency to onset. This is likely due to sluggish and highly variable uptake of this strongly hydrophilic compound (clogP = −2.18). At high AG concentrations (range 200 mM − 300 mM) latency onset was reduced but seizures continued to progress and became lethal. Thus, although the concept of GAD inhibition is attractive and relevant, AG administration in zebrafish is accompanied with practical issues that limit its applicability as a model for drug screening.

*In vitro* studies have shown that AG is a weak inhibitor of GAD with a Ki value of about 50 mM. Conversely, 2-keto-4-pentenoic acid (KPA), the *in vivo* occurring deaminated metabolite of AG, has a much stronger inhibitory effect with a Ki of 10^−6^ M. Moreover, in mice, ED50 values for seizure induction after intracerebroventricular (icv) administration is 14.5 µg/kg for KPA compared to 375 µg/kg for L-AG and 804 µg/kg for D-AG, respectively^37^.

Therefore, in this study, we synthesized ethyl ketopentenoate (EKP), a lipophilic ethyl ester of KPA, and tested its ability to induce seizures in 7 days post-fertilization (dpf) zebrafish larvae. Epileptiform activity was detected by locomotor tracking, local field potential and neuroluminescence recordings. For the latter, a novel assay was set up based on a transgenic luminescent zebrafish line. In addition, commercially available ASDs were evaluated for their ability to inhibit EKP-induced hyperactivity. We conclude that EKP is more effective than AG in inducing refractory seizures and could serve as a high-throughput model for the discovery of novel ASDs.

## Results

### *gad* expression during zebrafish development

**Figure 1:**
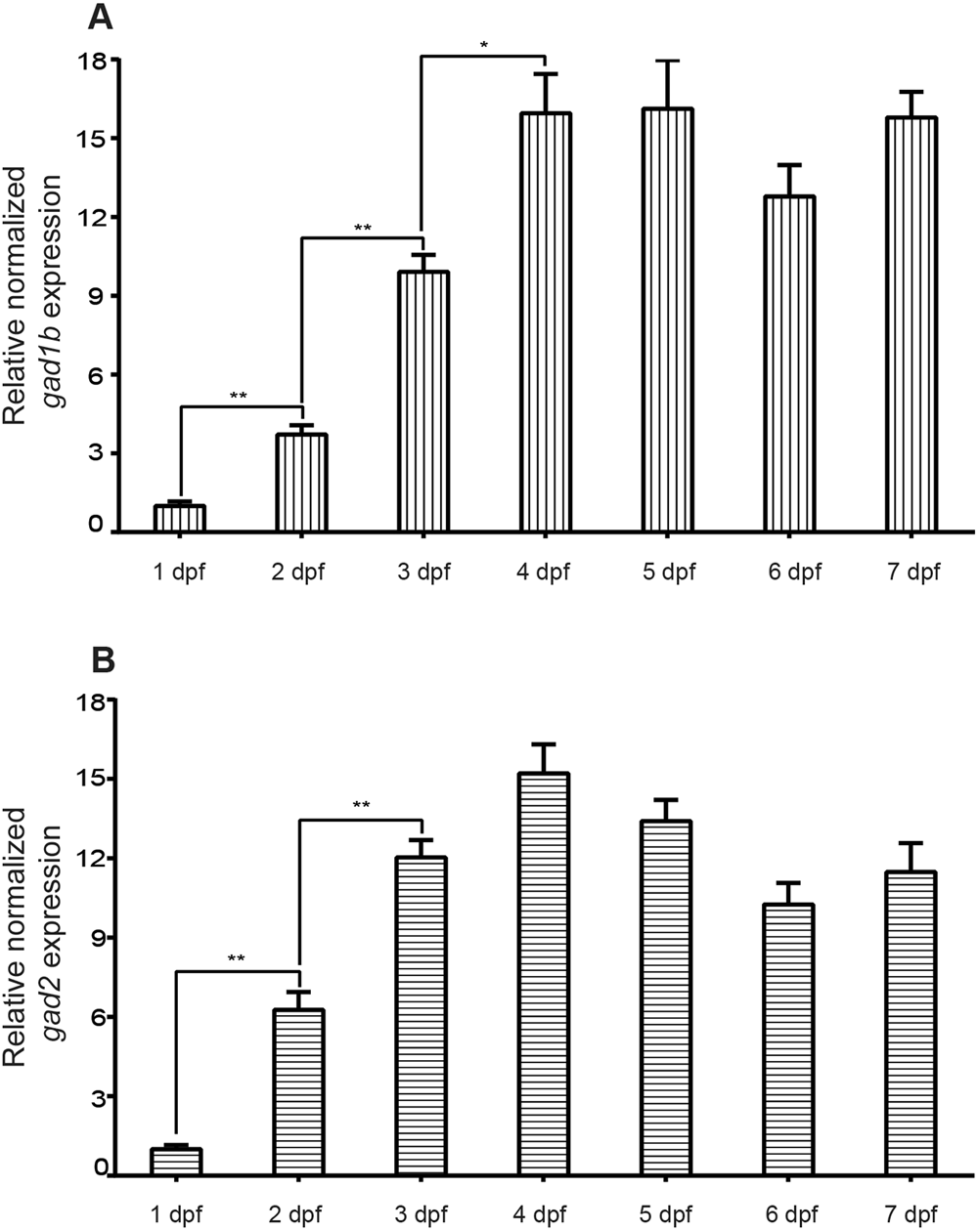
Developmental *gad1b* and *gad2* expression by qPCR. Gene expression levels were quantified relative to reference gene elongation factor 1 alpha (*elfα*), and 18s ribosomal RNA (*18s*) expression by ΔΔCq method. Zebrafish larvae samples from 1–7 dpf are shown. Results for each gene are expressed as mean ± s.e.m. of three experiments performed in triplicate. p-values calculated using Student’s unpaired t-test (*p ≤ 0.05). (**A**) Developmental expression of *gad1b*. (**B**) Developmental expression of *gad2*.

qPCR showed that both *gad1b* and *gad2* were expressed in zebrafish larvae between 1 dpf and 7 dpf (Fig. 1). Expression was low at 1 dpf, then significantly increased during the first 3 days of development (p ≤ 0.05) to remain constant at later stages (4–7 dpf, p > 0.05).

### Validation of larval zebrafish EKP seizure model

#### Locomotion activity and survival rate

Zebrafish larvae at 7 dpf were exposed to various concentrations of EKP (200 μM-800 μM) to identify an optimal working concentration for induction of seizure-like activities. Locomotor activities of both VHC- and EKP-treated larvae were monitored and quantified by an automated video-based behavioral tracking system.

**Figure 2:**
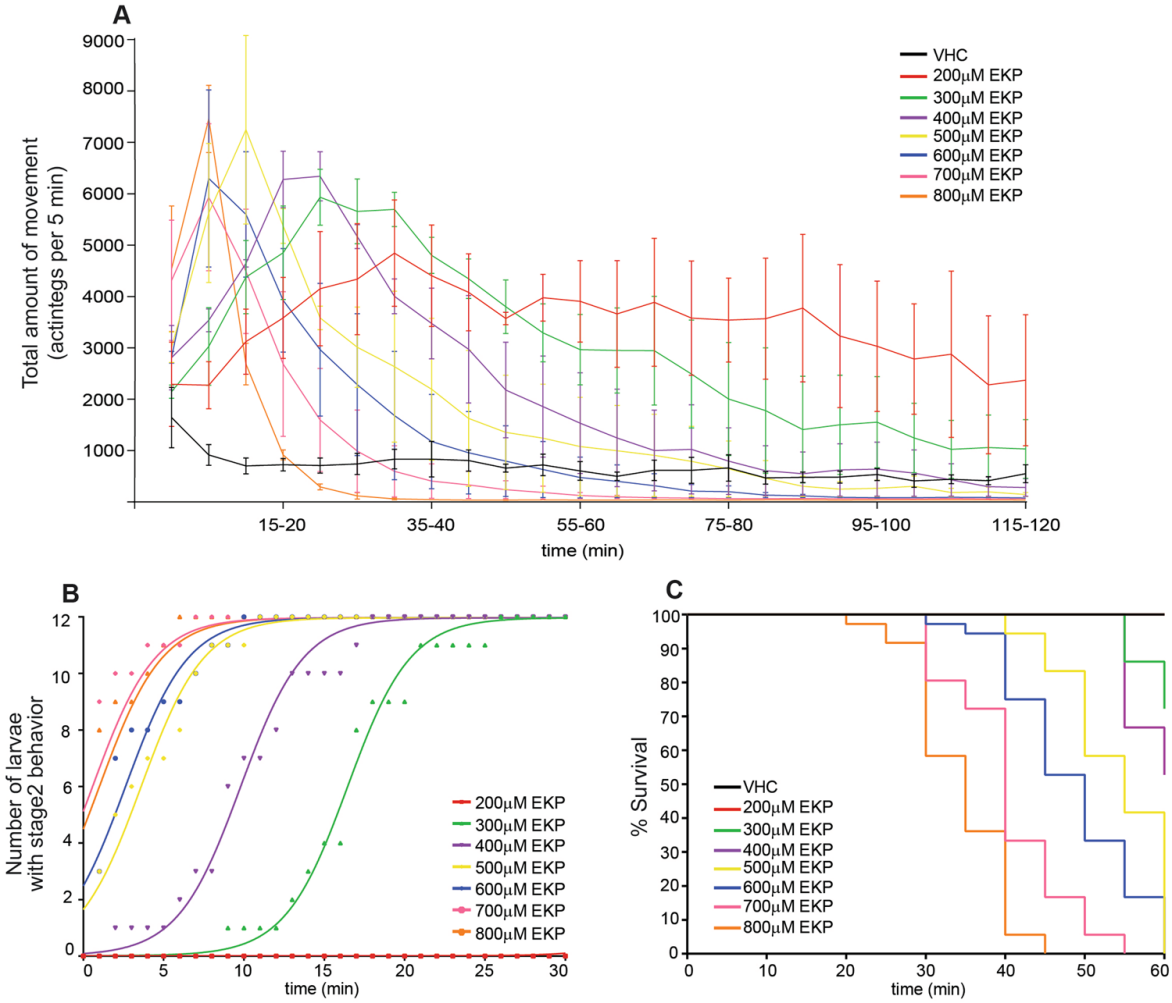
Behavioral profile of zebrafish larvae exposed to EKP. (**A**) Locomotor behavior of 7 dpf larvae treated with EKP (200 μM–800 μM) as compared to VHC-treated larvae. The data on the Y-axis refer to the average total movement of larvae per 5-minute (min) intervals. Results shown were pooled from four independent experiments with 12 larvae per experiment that were individually analyzed. Error bars represent s.d. (**B**) Phase 2 seizure-related behavior observed in zebrafish exposed to EKP. Zebrafish larvae at 7 dpf were incubated with medium containing 200 μM–800 μM EKP for 30 min. The total number of larvae displaying phase 2 behavior was added up per minute interval. Each group represents a total of 12 larvae. (**C**) Kaplan-Meier cumulative survival curve of 7 dpf larvae during exposure to 200 µM–800 μM EKP or VHC (n = 36 for each group).

VHC-treated larvae swam infrequently in a small dart-like manner and displayed baseline activities. Larvae exposed to EKP exhibited increased activity compared to VHC group in general with three different phases of seizure-related locomotion (Fig. 2A, statistical analysis see Table S2).

A first phase was characterized by an increasing number of bursts of hyperactivity and agitation. At EKP concentrations ≥300 μM this was followed by a second phase in which hyperactivity reached a plateau. At this point larvae also tended to swim more rapidly in a corkscrew-like manner accompanied with entire body jerking and twitching. In a third phase, the period of increased motility was followed by an irreversible decline of the total larval movement at concentrations between 300–800 μM. This was due to loss of body posture and paralysis (as observed under a microscope), and finally larval death (Fig. 2A). Onset and duration of each phase were clearly dose-dependent. As 400 μM EKP induced a robust increase of locomotor activity without inducing any larval death during the first 30 min (Fig. 2B,C), this concentration was used for further validation and pharmacological characterization of the model.

#### Local field potential (LFP) recordings and *c-fos* expression

**Figure 3:**
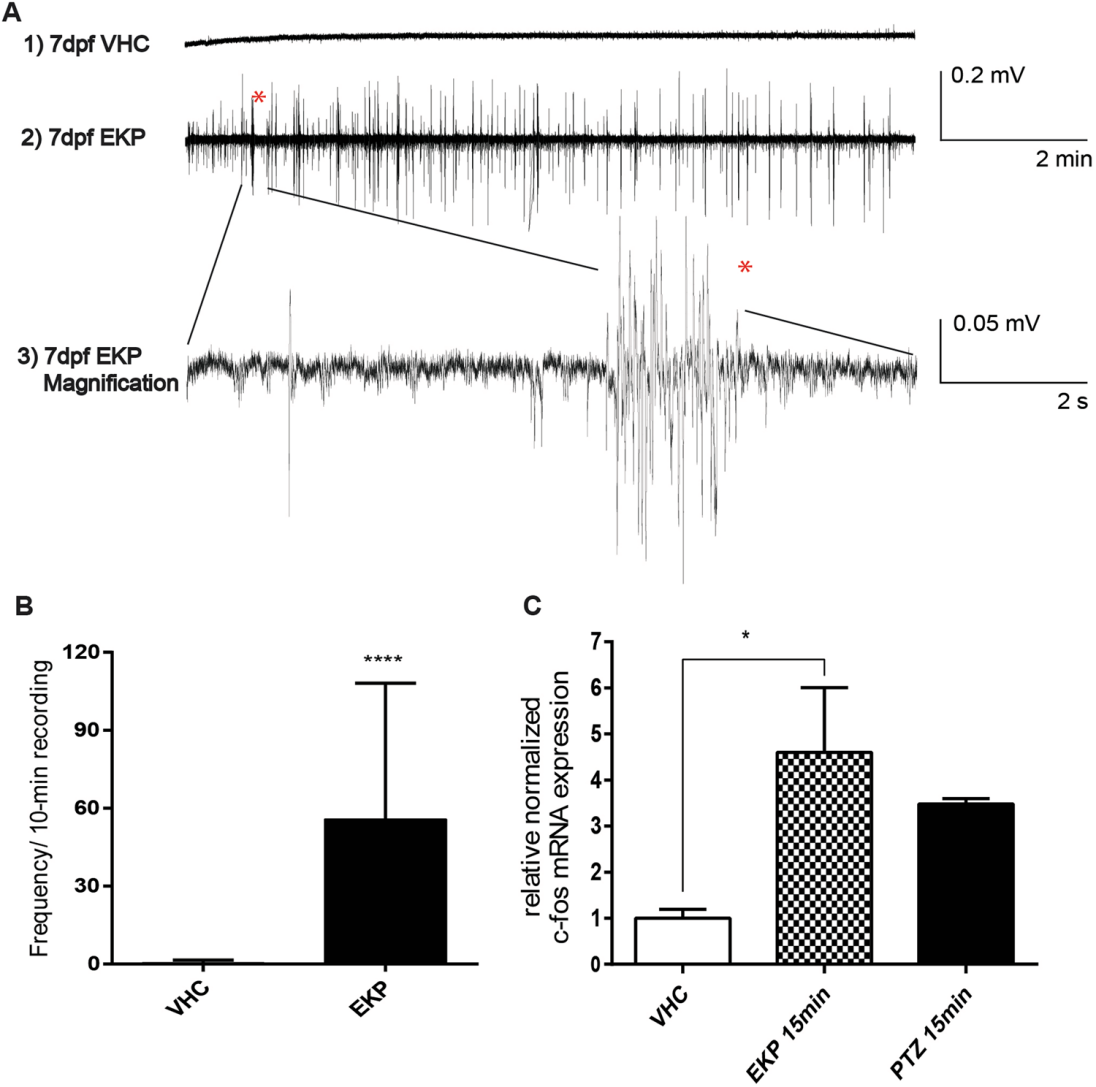
EKP induces abnormal electrographic activities in the optic tectum and *c-fos* expression in brain of 7 dpf larvae. (**A**) Fragments of representative recordings from 7 dpf larva treated with VHC (A1) or 15 min after application EKP (400 µM) (A2). Bottom trace (A3) shows a high-resolution magnification of the epileptiform events indicated above. (**B**) Number of epileptiform events in VHC and EKP treated larvae: VHC 0.4857 ± 0.1804 vs EKP 55.76 ± 6.872 events/10 min recording. The number of recordings analyzed were: VHC (n = 35) and EKP (n = 58). Statistical analysis was performed using Student’s unpaired t-test. Values that were significantly different compared to VHC were indicated with ****p ≤ 0.0001. Error bars on all graphs represent s.d. (**C**) EKP-induced seizure upregulated *c-fos* expression in 7 dpf zebrafish larvae as assessed by qPCR (PTZ was used as positive control). *c-fos* expression is normalized to the housekeeping gene *ef1α* and *β-actin 1*. The data were analyzed using the ΔΔCq method and reported as mean ± s.e.m. of three experiments performed in triplicate.

In order to confirm that 400 μM EKP-treated zebrafish larvae displayed abnormal brain activity, LFP recordings were performed in the optic tectum of both VHC- and EKP-treated 7 dpf larvae. 7 out of 35 VHC-treated larvae showed some epileptiform-like activity while multiple epileptiform events were found in all EKP-treated larvae (58/58) (Fig. 3A,B). In EKP-treated animals the mean frequency was 55.76 ± 6.87 events/10 min versus 0.48 ± 0.18 events/10 min in VHC-treated ones (Fig. 3B). Similarly to PTZ treated animals, *c-fos* expression was significantly increased in EKP-treated larvae compared to VHC-treated ones (Fig. 3C).

#### Neuroluminescence recordings

**Figure 4:**
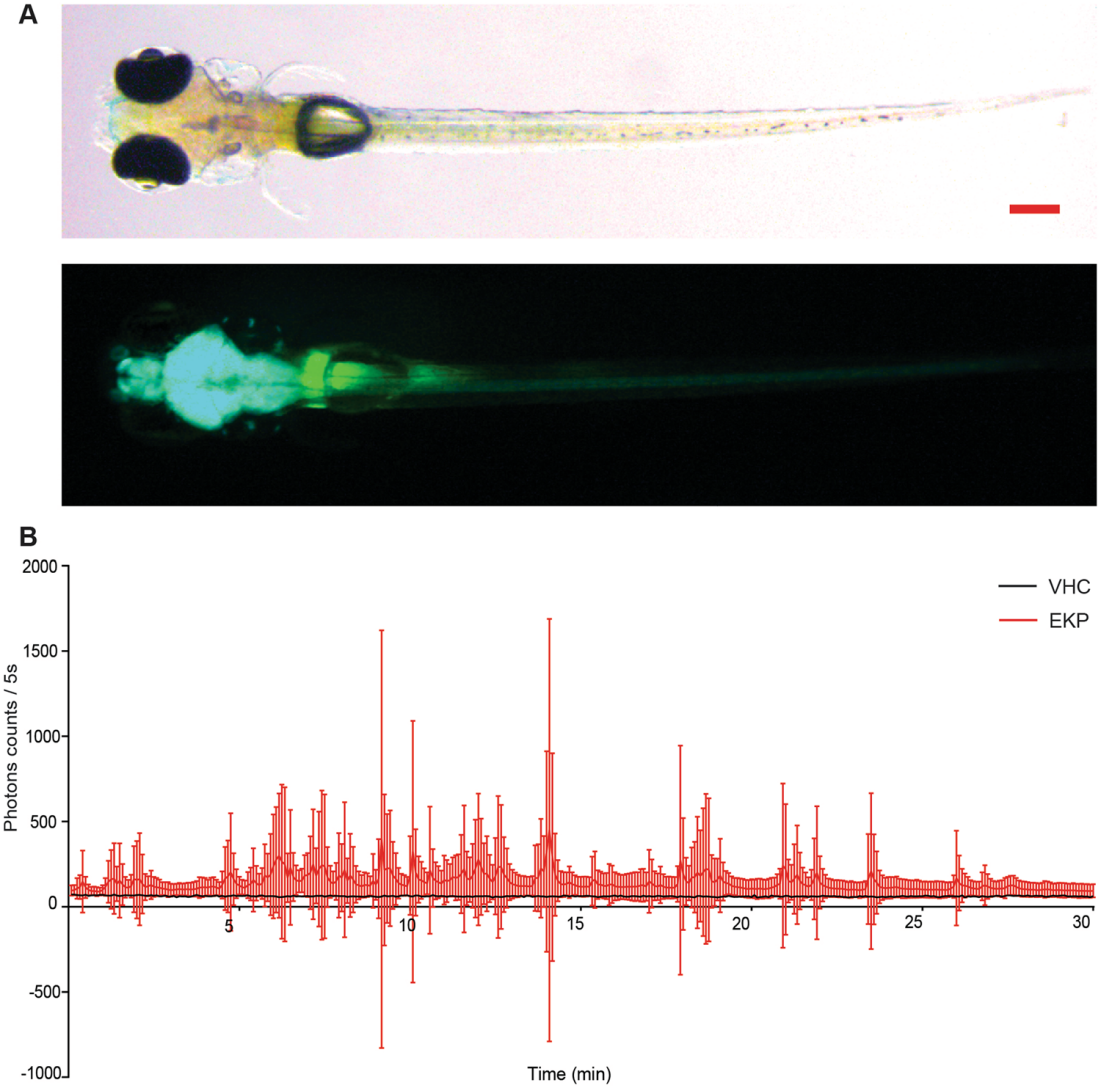
Neuroluminescence recording of *Tg(elavl3:GA)* zebrafish exposed to EKP. (**A**) Bright field (upper) and fluorescence (lower) micrographs of 7 dpf *Tg(elavl3:GA)* larva (scale bar: 0.20 mm) (**B**) Photon emission of 7 dpf larvae exposed to VHC (black line) or EKP (400 µM) (red line), after a 20 h incubation to CLZN-h (40 µM) exposure during a 30-min recording period. The total photon emission (y-axis) is counted per 5-second (sec) interval (x-axis). Results are expressed as mean ± s.d. of 18 independent experiments. For each experiment a group of three larvae was used.

Next, neuronal activity was assayed in freely behaving zebrafish using the luminescent approach as reported by Naumann *et al*.^38^. Transgenic zebrafish *Tg(elavl3:eGFP-apoAequorin(GA)﻿)*expressing GFP-apoAequorin under the control of the *elavl3* promoter were generated (Fig. 4A) and neuroluminescent signals were recorded for 30 min in VHC- and EKP-treated animals. In the VHC-treated group the averaged aequorin light signal remained relatively stable throughout the experiment. In EKP-treated larvae the averaged light signal was increased and displayed large fluctuations over time (Fig. 4B), resembling the LFP recordings in the same group. Hence, *in vivo* neuroluminescence clearly allows to detect and study abnormal brain activity occurring in freely behaving animals.

### Pharmacology activity

Next, 14 commercially available ASDs were evaluated for their ability to prevent EKP-induced epileptiform activity. Larvae were pre-incubated for 18 hours (h) with the indicated ASD at its maximum tolerated concentration (MTC). Epileptiform activity was scored with behavior tracking, LFP and neuroluminescence recordings.

**Figure 5:**
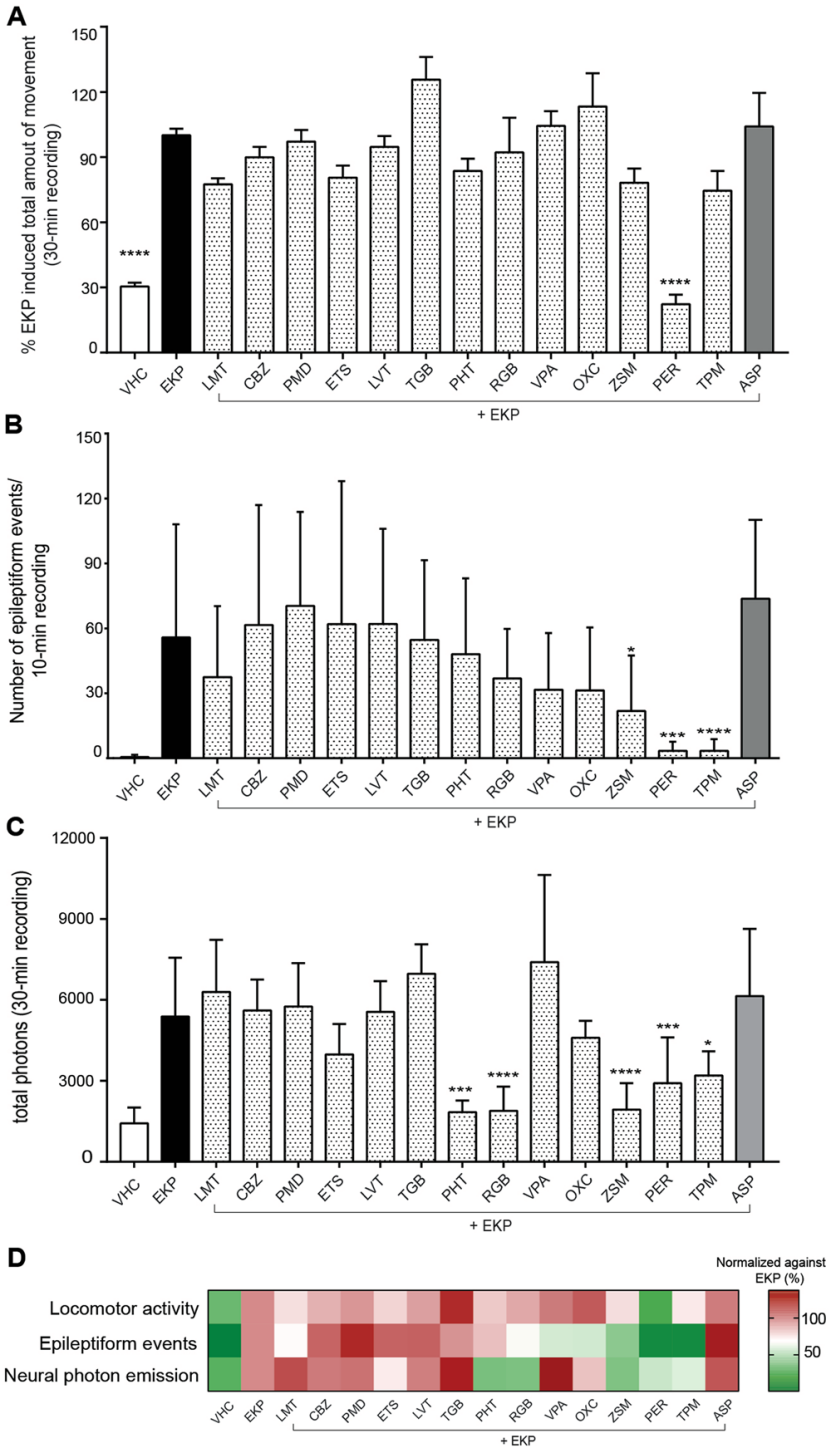
Quantitative analysis and comparison of locomotor activity, electrographic activity and neuroluminescence of larvae treated with EKP in combination with/without ASDs. (**A**) Larval locomotor activity within a 30-min recording normalized to EKP control (set at 100%). Results are shown as mean ± s.e.m. of 12 experiments (recordings of individual larvae) performed in triplicate. (**B**) Number of epileptic events within a 10-min recording (mean ± s.d.). Number of recordings analyzed were: VHC (n = 35), EKP (n = 58), LMT (n = 13), CBZ (n = 11), PMD (n = 14), ETS (n = 18), LVT (n = 14), TGB (n = 13), PHT (n = 14), RGB (n = 16), VPA (n = 14), OXC (n = 13), ZSM (n = 15), PER (n = 14), TPM (n = 16) and ASP (n = 12). (**C**) Number of photons emitted within a 30-min recording. Results are expressed as mean ± s.d. of at least 5 independent experiments. For each experiment a group of three larvae was used. All results (A, B, C) were analyzed using one-way ANOVA. Significant differences compared to EKP group are marked with *, **, ***, **** (p ≤ 0.05, p ≤ 0.01, p ≤ 0.001 and p ≤ 0.0001 respectively). (**D**) Heat map comparison of larval responses against EKP after different ASD treatment as examined by locomotor tracking, electrographic activity recording and neuroluminescence measurements. Data from ASD-treated larvae were normalized against results of the EKP-treated groups.

In case of behavior tracking, an ASD was considered to be active or slightly active if at least two time points or only one time point, respectively, showed a statistically significant decline in total movement in comparison with the EKP control group (Fig. S2)^11^. Based on these criteria, perampanel (PER) and zonisamide (ZSM) were identified as active compounds with PER as the only ASD that significantly prevented EKP-induced locomotion. Phenytoin (PHT) and topiramate (TPM) were identified as slightly active compounds (Fig. 5A).

LFP recordings revealed that ZSM, PER and TMP significantly attenuated the number of epileptiform discharges generated by EKP (Fig. 5B). Other ASDs were not active. For representative LFP recordings of EKP-treated larvae in combination with (or without) ASD pretreatment see Fig. S3.

For the neuroluminescent assay the cumulative amount of photons emitted during the 30-min EKP exposure period was quantified for VHC- and ASD-pretreated animals. ASDs were also tested at their MTC except for ETS and TGB which were evaluated at lower concentrations of 250 µM and 50 µM, respectively, due to toxic side-effects when co-incubated with CLZN-h. As shown in Fig. 5C, EKP-induced neuronal activity was significantly diminished in PHT, RGB, ZSM, PER and TPM pre-treated animals.

### Homology modelling and ligand docking

**Figure 6:**
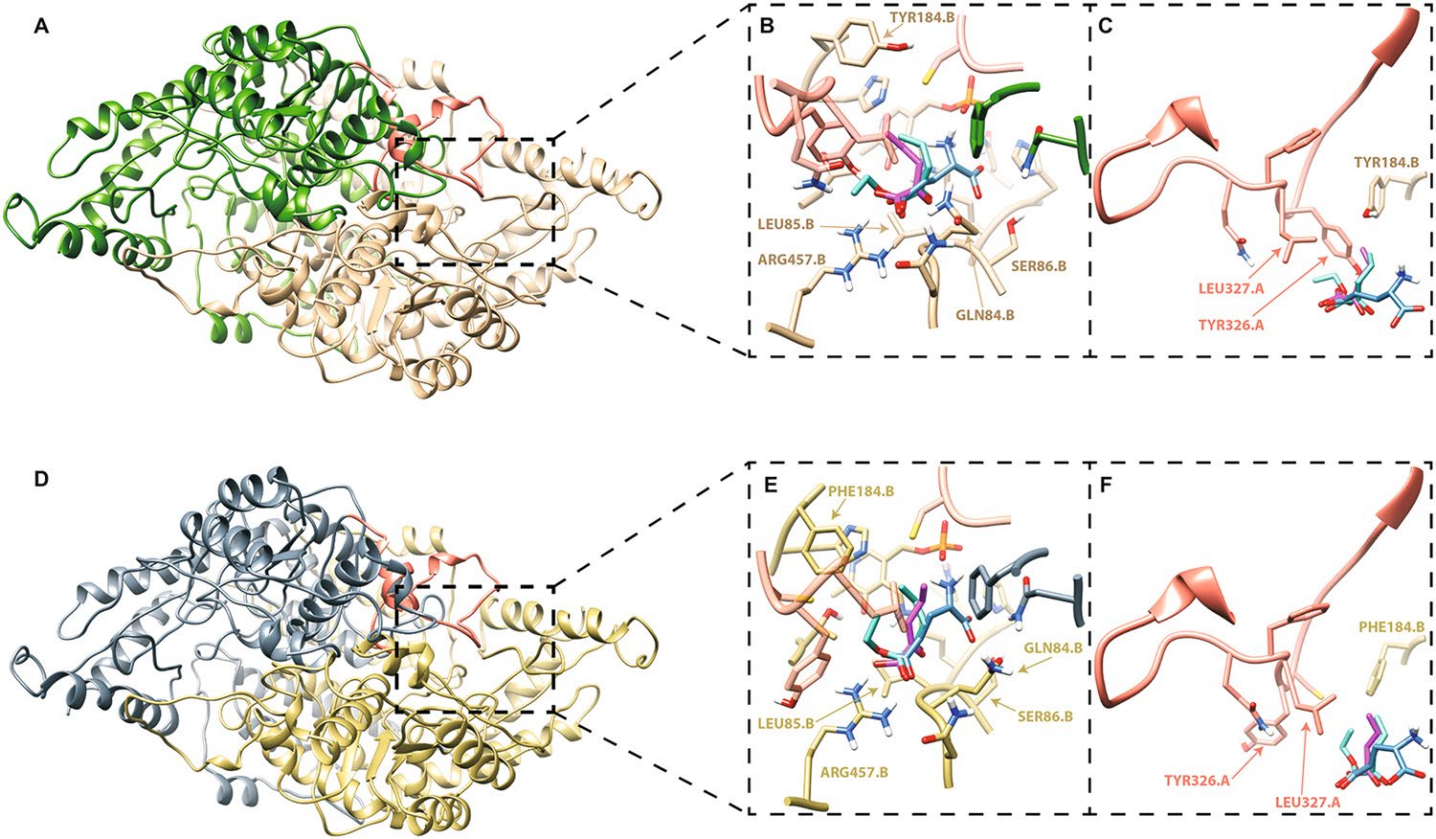
Dimeric models after refinement of the flexible loop (pink) for gad67 (**A**) and gad65 (**D**). Close-up on the active site with superimposed EKP (cyan), KPA (purple) and glutamate (blue) for gad67 (**B**) and gad65 (**E**). Important amino acids for interaction with the carboxyl-group are labeled. Zoom on the interactions of ligands with residues in the flexible loop in for gad67 (**C**) and gad65 (**F**).

The absence of 14 amino acids in the crystal structure for the human GAD65 (2OKK) is indicative of a flexible region in the protein. Therefore, the presence of a flexible loop was hypothesized by analyzing the superposition between the homology models and the human crystal structures. Final dimeric models after refinement of this flexible loop are shown in Fig. 6A and C for gad67 and gad65 respectively, demonstrating that the two subunits are intertwined as a result of an induced fit of the flexible loop (colored in pink).

For both final gad67 and gad65 dimeric models, the glutamate binding mode was compared to the ones of docked KPA and EKP. The binding affinity and the Ki values calculated by Autodock are reported in Table 1 for each of the complexes. Since values for the monomers are not within the range that is in agreement with observed *in vivo* effects, the obtained Ki values confirm the hypothesis of dimerization. A close up of studied inhibitors and the native glutamate in the active site of gad67 and gad65 is shown in Fig. 6B and E respectively, demonstrating that the spatial binding mode of both ligands overlaps with the native binding of glutamate. It also indicates the importance of the carboxyl group (or ester group in EKP) that is engaged in close interactions with GLN84.B, LEU85.B, SER86.B and ARG457.B in both enzymes. The fact that EKP binds with slightly less affinity, could be explained by the presence of an ester group whereas KPA has a carboxyl group that has more favorable electrostatic interactions with ARG457.B.

**Table 1:** Binding affinity (BA) in Kcal/mol and the Ki values in µM resulting from docking of GLU (glutamate), EKP and KPA in both monomeric and dimeric models of enzymes gad67 and gad65 of *Danio rerio*.

**Ki**	gad67 (µM)		gad65 (µM)	
	Monomer	Dimer	Monomer	Dimer
**GLU**	881.4	29.1	871.9	30.1
**EKP**	1110.6	45.8	1163.0	55.2
**KPA**	749.3	35.6	820.2	41.4
**BA**	**gad67 (kcal/mol)**		**gad65 (kcal/mol)**	
**GLU**	−4.18	−6.20	−4.18	−6.18
**EKP**	−4.04	−5.93	−4.01	−5.82
**KPA**	−4.28	−6.08	−4.22	−5.99

EKP (values in bold) shows similar results in comparison to GLU and KPA.

The importance of the dynamic loop (ALA307.A – PHE354.A) is highlighted by the extra interactions with both inhibitors as shown in Fig. 6C and F. At position 325.A the glycine is in gad67 replaced by cysteine gad65 which displaces TYR356.A out of the active site in gad65. In addition, the active site contains a PHE184.B in gad65 instead of a more polar TYR184.B in gad67. Both of these relatively small differences account for more extensive hydrophobic interactions to occur in the active site of gad65 compared to gad67, that are slightly withdrawing ligands from the crucial ARG457.A towards the hydrophobic part of the active site. In gad67 however, a spatially more favorable interaction is observed between the carboxyl moiety of ligands and ARG457.B, explaining the slightly improved Ki for ligands in the gad67 isozyme compared to gad65.

## Discussion

In this study we provide evidence that EKP acts as a strong inducer of epileptiform activities in zebrafish larvae. Compared with AG^13^, EKP induced hyperactivity at a 100-fold lower concentration. In addition, hyperactivity occurred more synchronously and with a fast onset. We also found a clear increase of epileptiform discharges in EKP-treated larvae that matches well with the outcome of the locomotor assay and the *c-fos* expression study.

Moreover, also a neuroluminescent approach was used to investigate the proconvulsant effect of EKP. The genetically modified larvae used to this end specifically express a Ca^2+^ sensor that generates bioluminescent signals upon strong neural activation^38^. We found that EKP exerted a dramatic effect on neuronal brain activity, as seen from the emission of large waves of neuroluminescence that could easily be measured and quantified.

From the experimental work it also became clear that the neuroluminescence-dependent assay offers several advantages over LFP recordings: (i) the larvae can freely move and hence there is no need to embed or paralyze them, (ii) recordings do not depend on the correct positioning of a needle that causes inherently interexperimental inconsistencies, (iii) brain activity is assessed as a whole as opposed to electrode recordings that monitor only a very small part of the brain, and (iv) long-term recordings are possible, which might be essential for zebrafish seizure models with infrequent and conditional seizure activities. Furthermore, the method is also compatible with future simultaneous live and video-based recordings of movement and epileptiform activities as an equivalent to clinical video-EEG monitoring.

Protein docking revealed that both KPA and EKP exhibited similar GAD binding affinity as glutamate. The interaction of the carboxyl (in KPA and glutamate) or ester group (in EKP) of these ligands with an arginine residue in the active site contributed strongly to protein binding. The hypothesis that dimers are formed and that a dynamic loop is present correlated with the observed difference in inhibition constants between the monomeric and dimeric models. Two of the mutations in the active site (Phe184 vs Tyr184 and Gly325 vs Ser325) could explain the small difference in affinity of the inhibitors towards gad65 and gad67, however altogether it appears that the proconvulsant activity observed in the different assays can be attributed to direct inhibition of both enzymes.

Further, we evaluated the efficiency of commonly used ASDs representing multiple mechanism of actions (MOA) to reduce the epileptiform zebrafish phenotype induced by EKP as assessed by the three different assay tools. To make a straightforward comparison, a heat map was generated that provides a visual summary of the EKP-related responses upon treatment with different ASDs. Total number of movements, epileptic events and photons emitted after ASDs pretreatment were normalized against the EKP-treated group (Fig. 5D). Overall, EKP-treated larvae responded poorly to most of the tested ASDs suggesting that EKP-induced seizures in zebrafish are treatment-resistant. Despite the fact that EKP is a potent inhibitor of GAD leading to GABA depletion no correlation could be found between the anti-seizure response and a specific MOA of different ASDs. This contrasts with a number of widely used seizure models utilizing GABA_A_ inhibiting compounds (e.g. PTZ, picrotoxin or bicuculline) which are most sensitive to GABAergic ASDs (e.g. barbiturates or benzodiazepines). Consequently, our observations could indicate that the EKP model does not show bias for any particular MOA of existing ASDs and may potentially lead to the discovery of mechanistically novel anti-seizure compounds.

Nevertheless, we observed that three ASDs, namely PER, ZSM and TPM, exhibited consistent reduction in epileptiform events. Of note, TPM as well as PHT were only slightly active in the locomotor activity assay. Additionally, PHT and RGB were found to be active in the neuroluminescence assay, but did not show significant effects in LFP recordings. In fact, certain ASDs may have preferential effects against secondary generalization and spread of seizures, which had been observed in rodent models like the amygdala kindling^39^. The observed difference may also stem from the fact that neuroluminescence monitor whole brain activity, whereas LFP measurements detect signals generated from a small population of optic tectal neurons. As a result, LFP-based assays tend to be less sensitive in detecting signals occurring in brain locations that are distant from the recording spot.

Thus, neuroluminescence offers a window of opportunity to identify compounds that would have passed unnoticed by standard electrophysiological methods. Furthermore, neuroluminescence assay may be able to detect seizure-like activity that is not associated with clear-cut convulsive behaviors in zebrafish, e.g. non-convulsive seizures. Since our study represents the first comprehensive evaluation of ASDs efficacy in a convulsant-induced seizure zebrafish model using a neuroluminescence readout, the model will be further tested with the criteria of reliability and usefulness using other convulsant compounds. This will provide more information about its general applicability and detection rates of false positive and negative compounds. In addition, further developments are possible as it feasible to combine the synchronous recording of bioluminescent signals and locomotor behavior of the freely moving larvae^38^. Moreover, zebrafish expressing genetically encoded calcium indicators (GCaMPs) have successfully been used to monitor in real-time whole brain 3D neuronal activity in immobilized zebrafish larvae^40^. When compared to the bioluminescent approach that does not provide any spatial information on the emitted light signal, this fluorescence-based method would possibly allow to localize the EKP-induced seizures and discern whether they are focal or not, and investigate further into detail the anti-seizure activity of a few selected compounds of interest.

Epilepsy is a very heterogeneous disease and acute preclinical seizure models typically do not mimic any particular epilepsy syndrome, but rather reflect certain seizure types (e.g. MES, PTZ or 6 Hz). Yet, it can be argued that animal models of seizures have shown generally high predictive validity for a therapeutic drug response in patients. Thus, any seizure model does not need to be a perfect replication of the clinical condition, but it is important that the validation provided for a given model is “fit for purpose”^41^.

We have demonstrated for the first time a robust convulsive effect of EKP, a potent GAD inhibitor, in 7 dpf zebrafish larvae. EKP increased locomotor activity, induced epileptiform events and a corresponding increase in neuroluminescence, and increased synaptic activity-regulated *c-fos* expression.

EKP-induced seizures were similar to those caused by AG, but with much shortened seizure latency and a more synchronized seizure onset. However, it remains to be established if this model, like many other acute seizure models in both zebrafish and rodents, represent any particular epilepsy syndrome despite strong evidence that GAD has been implicated in pathophysiology of epilepsy. Our findings support the suggestion that the EKP zebrafish model is characterized by poor response to several existing ASDs and may become a useful addition to the armamentarium of animal models of drug resistant seizures. In fact, no single model has been validated for use to identify potential compounds effective against drug resistant seizures. Consequently, it is suggested that a battery of such models should be employed, thus enhancing the sensitivity to discover novel, highly effective ASDs^42^.

In conclusion, the zebrafish EKP model can therefore be used for screening of drug-like hits in an early step of the discovery of mechanistically novel ASD candidates. Furthermore, it is anticipated that by providing quantifiable whole brain-related Ca^2+^ signals in freely moving transgenic animals, the neuroluminescence technique might turn out to be of particular interest for future drug discovery strategies in the field of epilepsy and epileptogenesis.

## Materials and Methods

### Animals and maintenance

Adult zebrafish (*Danio rerio*) were maintained in a UV-sterilized rack recirculating system equipped with a mechanical and biological filtration unit and kept under a 14/10 hour light/dark cycle at the temperature of 27–28 °C and pH of 6.8–7.5. Water quality was monitored for pH, temperature, conductivity, ammonia, nitrite (SL1000 Portable Parallel Analyzer, Hach Instruments, USA) and nitrate levels (Tetra, Melle, Germany). Zebrafish were fed three times per day with flake food (TetraMin, Tetra, Germany) and Artemia (brine shrimp). Embryos were obtained via natural spawning, then sorted and kept in petri dishes (92 × 16 mm, Sarstedt, Nümbrecht, Germany) at 28 °C in a Peltier-cooled incubator (IPP 260, Memmert, Schwabach, Germany) in embryo medium (Danieau’s solution: 1.5 mM HEPES, 17.4 mM NaCl, 0.21 mM KCl, 0.12 mM MgSO_4_, and 0.18 mM Ca(NO_3_)_2_ and 0.6 μM methylene blue). For the generation of the *Tg*(*elavl3:eGFP-apoAequorin*) line, the *mitfa* ^−/−^ (nacre) strain was used. This strain lacks body pigmentation and hence the fluorescent/neuroluminescent signal is easier to observe. For all other experiments 1–7 dpf larvae of the AB strain were used.

All zebrafish experiments were approved by the Ethics Committee of the University of Leuven (Ethische Commissie van de KU Leuven, approval number ECD P101/2010) and by the Belgian Federal Department of Public Health, Food Safety & Environment (Federale Overheidsdienst Volksgezondheid, Veiligheid van de Voedselketen en Leefmileu, approval number LA1210199). All procedures were carried out according to the Declaration of Helsinki and conducted according to the guidelines of the European Community Council directive 86/609/EEC.

### Compound treatment

Ethyl ketopentenoate (EKP) was synthetized according to a procedure described in supplementary materials. Pentylenetetrazol (PTZ), lamotrigine (LMT), carbamazepine (CBZ), primidone (PMD), ethosuximide (ETS), levetiracetam (LVT), valproate (VPA), oxcarbazepine (OXC), zonisamide (ZSM), topiramate (TPM) and aspirin (ASP) were purchased from Sigma. Other compounds used were: phenytoin (PHT) (Acros), tiagabine (TGB) (Chemos), perampanel (PER) (Eisai), retigabine (RGB) (Valeant Pharmaceuticals/GlaxoSmithKline) and coelenterazine-h (CLZN-h) (NanoLight® Technologies). All compounds were dissolved in dimethyl sulfoxide (DMSO) and kept as a stock at −20 °C or −80 °C. Stock solutions were further diluted in embryo medium for locomotor activity assay and local field potential recordings, or E3 medium (5 mM NaCl, 0.17 mM KCl, 0.33 mM CaCl_2_ and 0.33 mM MgSO_4_) for neuroluminescence recording to achieve a final DMSO concentration of 1% w/v. As vehicle control (VHC) 1% w/v DMSO in embryo or E3 medium was used.

### Generation of *Tg(elavl3:eGFP-apoAequorin)* line

A transgenic fish line *Tg*(*elavl3:eGFP-apoAequorin*) was generated with the pan-neuronal *elavl3* promoter that drives the expression of a fusion of eGFP and apoAequorin. Briefly, the *elavl3* promoter (a gift from Dr. Florian Engert, Harvard, USA) and the coding sequence for the eGFP-apoAequorin fusion protein^43^ (a gift from Dr. Ludovic Tricoire, Université Pierre/Marie Curie, France) were cloned into a gateway expression vector flanked with *tol2* recognition sites. To generate stable transgenic zebrafish, 20 pg of the *elavl3:eGFP-apoAequorin* plasmid DNA was co-injected with 100 pg *tol2* transposase mRNA into the cytoplasm of single cell stage fertilized nacre zebrafish embryos. Injected embryos were screened for eGFP expression at 2–5 dpf. eGFP positive individuals (F0) were grown to adulthood and out-crossed with nacre zebrafish. All the experiments with were performed with progeny (F3) from intercrossing stable F2 transgenics.

### Analysis of *gad1b* and *gad2* expression using qPCR

*A. RNA extraction and cDNA synthesis:* Total RNA was extracted from 12 embryos on 1 to 7 dpf using TRIzol (Life Technologies) according to manufacturer’s instructions. Following DNase (Roche) treatment, RNA was quantified by NanoPhotometer P330 (Implen). cDNA was synthesized from 1 μg of total RNA using random primers and SuperScript III reverse transcriptase (Invitrogen) according to the manufacturer’s instructions and further diluted 1:20.

*B. qPCR analysis:* For analysis of *gad1b* and *gad2* mRNA expression, 4 μl of each cDNA was amplified with Bio-Rad 20x PrimePCR assays (*gad1b* assay ID: qDreCID0019786; *gad2* assay ID: qDreCID0014650) and 2x SsoAdvanced Universal SYBR Green Supermix (Bio-Rad). H_2_O was used as none template control (NTC). qPCR reactions were performed with HardShell® Low-Profile Thin-Wall 96-Well Skirted PCR Plates (Bio-Rad) on CFX96 Touch Real-Time PCR Detection System (Bio-Rad) under cycling conditions according to the manufacturer’s protocol. Data generated by real-time PCR were compiled using CFX Manager Software (Bio-Rad). The *gad1b* and *gad2* transcripts were normalized against *ef1α* and *18 s* (primers: Table S1). The relative expression levels were quantified using the comparative Cq method (ΔΔCq). Amplification specificity was monitored by examining the final melting curve.

### Validation of larval zebrafish EKP seizure model

#### Locomotion activity

Larvae (6 dpf) in 100 µl VHC were arrayed individually in a 96-well plate (tissue culture plate, flat bottom, Falcon, USA) and kept for 18 h in the dark at 28 °C. Prior to tracking the following day (7 dpf) 100 μl of VHC or EKP stock solution was added to each well to obtain a range of EKP concentrations (200 μM-800 μM). The plates were placed in an automated video tracking device (ZebraBox^TM^ apparatus; Viewpoint, Lyon, France) and 5 min later the locomotor behavior of the larvae was monitored for 2 h in the dark at 28 °C. Locomotor activity was quantified using ZebraLab™ software (Viewpoint, Lyon, France) and expressed in “actinteg” units per 5-min interval. The actinteg value is defined as the sum of all image pixel changes detected during the time window.

Video recordings of individual wells were used between 0–30 min to assess the cumulative number of larvae exposed to the different EKP concentrations that had exhibited phase 2 seizures. These seizures are defined as rapid corkscrew-like accompanied with entire body jerking and twitching, which are easily distinguished from other behavioral abnormalities (for description of phase 1 and 3 seizures, see results).

#### Survival rate

Larvae (7 dpf) were arrayed individually in a 96-well plate, exposed to EKP (200 μM-800 μM) or VHC (control) and immediately scored under a stereomicroscope for lethality every 5 min for 1 h.

#### Local field potential (LFP) recordings

Larvae (6 dpf) in 100 µl VHC were arrayed individually in a 96-well plate and kept for 18 h in the dark at 28 °C. An equal volume of 800 μM EKP (2x solution) or VHC (control) was added to each well and hence larvae were incubated with 400 μM EKP for 15 min. Next, the treated larva was embedded ventral side down in 2% low melting point agarose bathed in artificial cerebrospinal fluid (ACSF, 124 mM NaCl, 2 mM KCl, 2 mM MgSO_4_, 2 mM CaCl_2_, 1.25 mM KH_2_PO_4_, 26 mM NaHCO_3_, and 10 mM glucose). A blunt glass electrode (soda lime glass, Hilgenberg, Germany) was pulled with DMZ Universal Puller (Zeitz, Germany) to an opening of 15–20 microns, filled with ACSF and placed on the skin above the optic tectum. The recordings were performed using WinEDR (John Dempster, University of Strathclyde, UK). Differential signal was band pass filtered at 0.3–300 Hz and digitized at 2 kHz via a PCI-6251 interface (National Instruments, UK). LFP recordings started each time exactly 2 min after the removal of the larva from the EKP solution (or VHC) and were continued for 10 min at room temperature (24 °C).

EKP-induced events were considered as epileptiform activity when their signal exceeded three times the baseline and lasted for minimum 100 msec, as described previously^44^. Electrophysiological recordings were analyzed in a blinded way using Clampfit 10.2 software (Molecular Devices, USA).

#### Analysis of *c-fos* expression in larval heads using qPCR

To measure the seizure-related changes in *c-fos* mRNA expression in the CNS, 12 larvae (7 dpf) were treated with either 20 mM PTZ, 400 μM EKP or VHC for 15 min and then rapidly decapitated. The heads were immediately processed for RNA isolation followed by cDNA synthesis as described for *gad1b* and *gad2*. qPCR analysis of *c-fos* mRNA expression using Bio-Rad 20x PrimePCR assays (*c-fos* assay ID: qDreCED0015103) was performed as described for *gad1b* and *gad2*, and normalized to the housekeeping gene *ef1α* and *β-actin 1* (primers, Table S1).

#### Neuroluminescence recording

*Tg*(*elavl3:eGFP-apoAequorin*) zebrafish larvae were visually screened for even GFP expression at 3 dpf. Selected larvae were incubated with VHC in 40 µM CLZN-h for 18–24 h in the dark at 28 °C^38^. Excess of CLZN-h was removed by washing larvae repeatedly in E3 medium afterwards. A group of 3 larvae was then transferred into a light-tight thermostated perfusion chamber (27–28 °C) containing either VHC or 400 µM EKP. The recordings started each time exactly 5 min after EKP exposure and were continued for 30 min. Photons emitted were detected by a photon-counting tube (Type H3460–04, Hamamatsu Photonics, Japan) that was positioned about 2 cm above the larvae. Light impulses were discriminated, prescaled and counted with a PC-based 32-bit counter/timer board (PCI-6601, National Instruments Corporation, Austin, TX, USA). The number of impulses occurring during a 5-sec time interval was monitored with custom-built software. Analysis of the bioluminescence recordings was done in a blinded way.

### Pharmacology of larval zebrafish EKP seizure model

**Figure 7:**
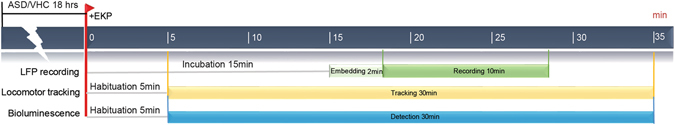
Schematic comparison of the experimental timelines as used for the locomotor tracking (yellow), LFP (green) and neuroluminescence recordings (blue). Key time points for each experimental protocol are indicated.

A schematic comparison of timelines of the different experiments is depicted in Fig. 7.

#### Maximum tolerated concentration (MTC)

Before performing pharmacological experiments, MTC of each ASD was determined as described previously^11^.

#### Effect of ASDs on EKP-induced locomotion activity

Larvae (6 dpf) were exposed to VHC or ASDs at their respective MTC for 18 h in the dark at 28 °C. Then, the larvae were treated with 400 µM EKP (or VHC) and the locomotion activity was monitored for 30 min (after 5 min of habituation) as described earlier. The average amount of movement per 5-min intervals was measured, quantified and normalized to the EKP control group for the entire 30-min tracking session or per 5-min interval.

#### Effect of ASDs on EKP-induced epileptiform discharges

Larvae (6 dpf) were exposed to VHC or ASDs at their respective MTC for 18 h in the dark at 28 °C. Then, the larvae were treated with 400 µM EKP (or VHC), embedded in agarose, and the LFP recordings were performed as described earlier.

#### Effect of ASDs on EKP-induced neuroluminescent events

eGFP-positive *Tg(elavl3:GA)* zebrafish larvae (6 dpf) were exposed to VHC or ASDs at their respective MTC (as determined in E3-CLZN solution) and 40 µM-h CLZN for 18 h in the dark at 28 °C. Larvae were then washed repeatedly in E3 medium and groups of three larvae were transferred into a light-tight thermostated perfusion chamber (27–28 °C) containing either VHC or 400 µM EKP. Recordings were performed as described earlier.

### Protein ligand docking

Using i-Tasser^45^, homology models for gad65 and gad67 were created for Uniprot sequences F1R9E8 and Q7ZUS3 and pdb structures 2OKK and 2OKJ as templates respectively^46^. Cα traces in dimer models are constructed with COTH^47^ starting from 2OKJ using 484 residues in each of the subunits (first 97 residues in gad65 and 101 in gad67 are omitted). For the ease of comparison, residue numbering in both models starts at 1. The SABBAC server^48^ and AMBER software^49^ were used for backbone reconstruction and final energy minimization respectively. Loop remodeling was performed with the loop refinement tool in USCF Chimera^50^. Finally, a short molecular dynamics simulation of 20 nanoseconds (nsec) was performed. Ramachandran plots were hereafter generated to verify the quality of the homology model^51^ (Fig. S1).

Flexible docking was performed using Autodock^52^ in one of the active sites of the dimeric homology models. A grid unit of 0.375 A was used with a 3-dimensional box having sides of 40 units. The grid box center for gad67 and gad65 was chosen to be (11.693, 17.627, −20.110) and (−6.749, 16.877, 132.140) respectively. Residues 108.A, 326.A, 327.A, 329.A, 347.A, 83.B, 84.B, 85.B, 86.B, 183.B, 184.B, 240.B, 297.B, 457.B were selected to be flexible. The Genetic Algorithm output was chosen in combination with the generation of 100 docked structures. The conformations located in the top cluster (with an rmsd-value of zero) were visually analyzed in USCF Chimera^50^. Within each gad, the conformations were superimposed to visualize differences in binding mode. Analysis of hydrogen bonds and hydrophobic interactions was performed by USCF Chimera and LigPlot+^53^. N6-(pyridoxal phosphate) lysine was parametrized based on empirical data for all docking and molecular dynamics simulations.

### Data analysis

Statistical analysis was performed using one-way or two-way ANOVA followed by Dunnett’s multiple comparison test with GraphPad Prism Version 7.0c (San Diego, CA).

### Data availability

All data supporting this work are included in this manuscript.

## Electronic supplementary material

Supplementary information
