# Supplementary material for: Inhibition of glutamate decarboxylase (GAD) by ethyl ketopentenoate (EKP) induces treatment-resistant epileptic seizures in zebrafish
Source: Sci Rep. 2017 Aug 3;7:7195. doi: 10.1038/s41598-017-06294-w (PMC5543107; doi:10.1038/s41598-017-06294-w)
Supplement: Supplementary file 1 — Supplementary information [file 41598_2017_6294_MOESM1_ESM.pdf]

Inhibition of glutamate decarboxylase (GAD) by ethyl ketopentenoate (EKP) induces treatment-resistant epileptic seizures in zebrafish

Yifan Zhang [1], Michiel Vanmeert [2], Aleksandra Siekierska[1], Annelii Ny [1], Jubi John [3#], Geert Callewaert [4], Eveline Lescrinier [2], Wim Dehaen [3], Peter A. M. de Witte [1, *], Rafal M. Kaminski [5, **]

[1] Laboratory for Molecular Biodiscovery, Department of Pharmaceutical and Pharmacological Sciences, KU Leuven, Leuven, Belgium.

[2] REGA institute for Medicinal Chemistry, Department of Pharmaceutical and Pharmacological Sciences, KU Leuven, Leuven, Belgium.

[3] Molecular Design and Synthesis, Department of Chemistry, KU Leuven, Leuven, Belgium.

[4] Department of Cellular and Molecular Medicine, KU Leuven, Leuven, Belgium.

[5] Neuroscience TA, UCB Biopharma, Braine-l'Alleud, Belgium

# Current address: Organic Chemistry Section, CSTD, CSIR-NIIST, Thiruvananthapuram-19, Kerala, India

* Author for correspondence: P.A.M. de Witte, Laboratory for Molecular Biodiscovery, Department of Pharmaceutical and Pharmacological Sciences, University of Leuven, Leuven, Belgium. Tel.: +32 16 32 34 32 ; E-mail: peter.dewitte@kuleuven.be

** Author for correspondence: R.M. Kaminski, Neuroscience TA, UCB Biopharma sprl, Avenue de l'Industrie, R9, B-1420 Braine-l'Alleud, Belgium. Tel.: +32 2 386 6499; E-mail: rafal.kaminski@ucb.com

Supplementary Material

Synthesis of ethyl ketopentenoate (EKP)

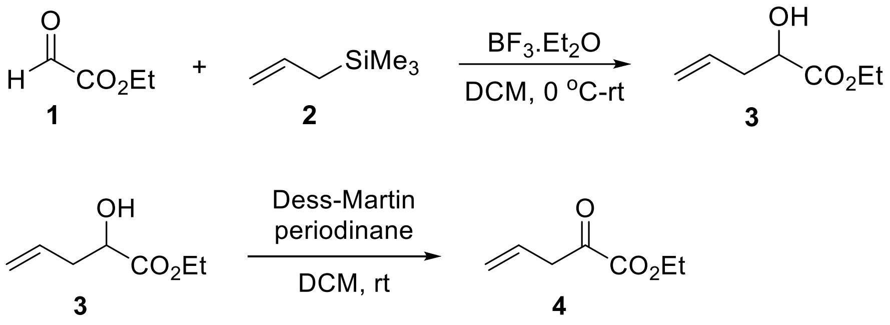

Ethyl ketopentenoate (EKP) (**4**) was prepared by adding dropwise boron trifluoride diethyl etherate (1.23 mL, 9.8 mmol) to a solution of ethyl glyoxylate (**1)** (1 g, 9.8 mmol) and allyltrimethylsilane (**2)** (2.23 g, 19.6 mmol) in dry CH_2Cl_2 (25 mL) at 0 °C, according to [1]. The solution was allowed to warm up to ambient temperature and was stirred for an additional 1.5 hrs. The reaction was quenched with a saturated aqueous solution of NH_4Cl and extracted with CH_2Cl_2 (3 x 20 mL). The combined organic extracts were washed with brine (50 mL), dried over $MgSO_4$, and concentrated by rotary evaporation to yield a crude mixture containing compound **3** as yellow oil. This crude mixture was used without further purification. Then, to the solution of compound **3** (500 mg, 3.5 mmol) in CH_2Cl_2 (20 mL), Dess-Martin periodinane (1.6 g, 3.8 mmol) was added at room temperature. After the completion of the reaction as seen on TLC, the mixture was washed with 20 mL of 1:1 10% $Na_2S_2O_3$: saturated aqueous $NaHCO_3$, followed by 20 mL of H_2O and brine. The organic layer was then dried with $MgSO_4$ and concentrated. Flash chromatography (hexane-ethyl acetate) provided ethyl ketopentenoate (**4**) as a light yellow oil (380 mg, 76%).

Data compound **3**: ^1H NMR (300 MHz, $CDCl_3$): δ 1.30 (t, J = 6.9 Hz, 3 H); 2.40-2.62 (m, 2 H), 2.83 (brs, 1 H), 4.20-4.31 (m, 3H), 5.12-5.20 (m, 2 H), 5.88-5.75 (m, 1H) ppm; Exact mass (HRMS, EI) calculated for $C_7H_{12}O_3$: 144.0786, found 144.0792.

Data ethyl ketopentenoate (**4**): ^1H NMR (300 MHz, $CDCl_3$):2 δ 1.35 (3H, t, J = 7.2 Hz), 4.31 (2H, q, J = 7.2 Hz), 5.24-5.28 (1H, m), 5.39-5.45 (1H, m), 6.22 (1H, d, J = 11.3 Hz), 6.71-6.84 (1H, m) ppm; ^{13}C NMR (75 MHz, $CDCl_3$):2 δ 14.0, 62.5, 112.3, 120.2, 129.8, 139.7, 162.0 ppm; Exact mass (HRMS, EI) calculated for $C_7H_{10}O_3$: 142.0630, found 142.0637.

Supplementary Figures

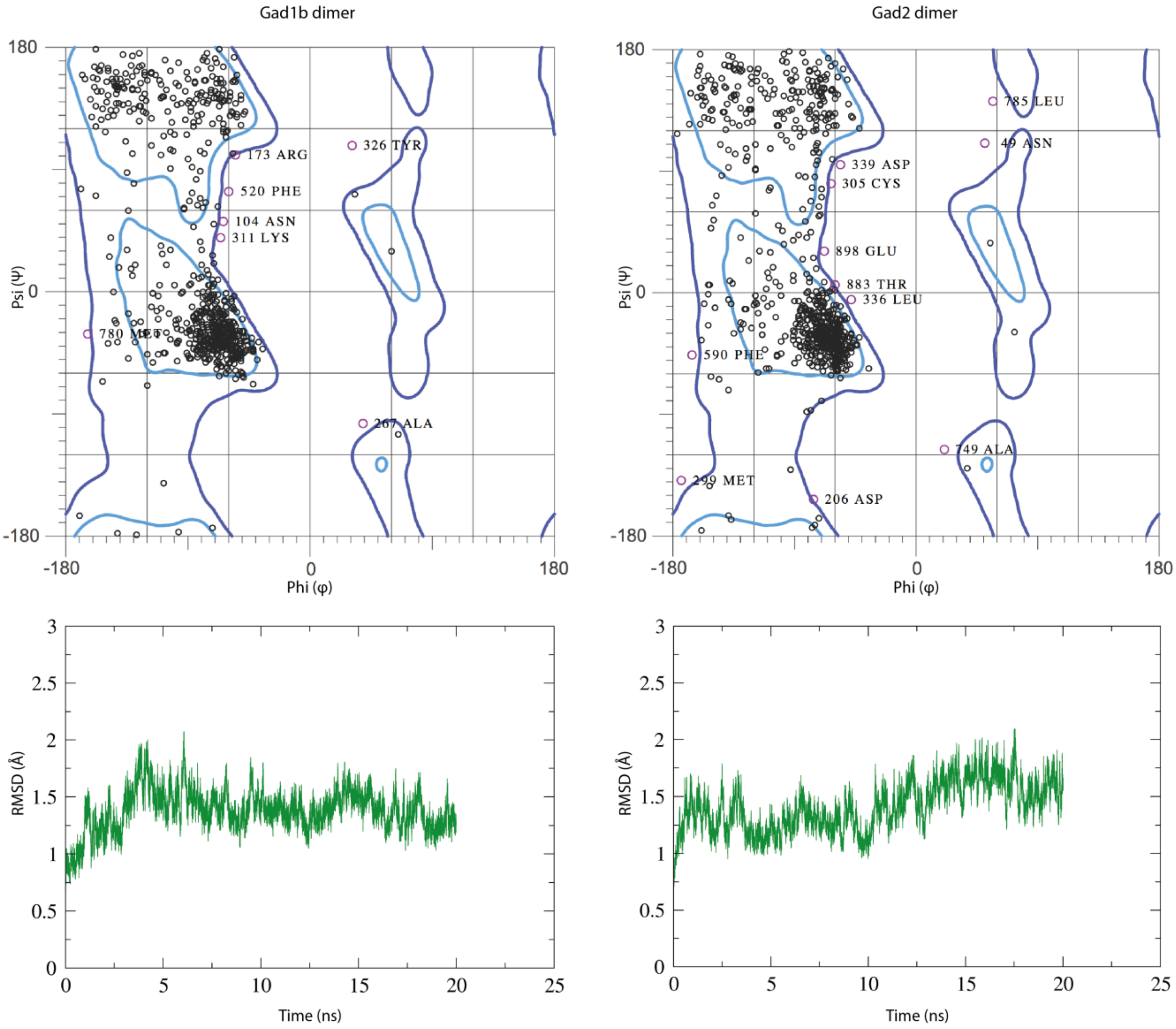

Fig. S1: **Ramachandran and RMSD plots for gad1b and gad2 dimeric models.** Gad1b dimer shows >99 % of residues in allowed regions. A stable RMSD was reached after 20 ns simulation. Gad2 shows >98% of residues in allowed regions. A stable RMSD was reached after 20 ns simulation.

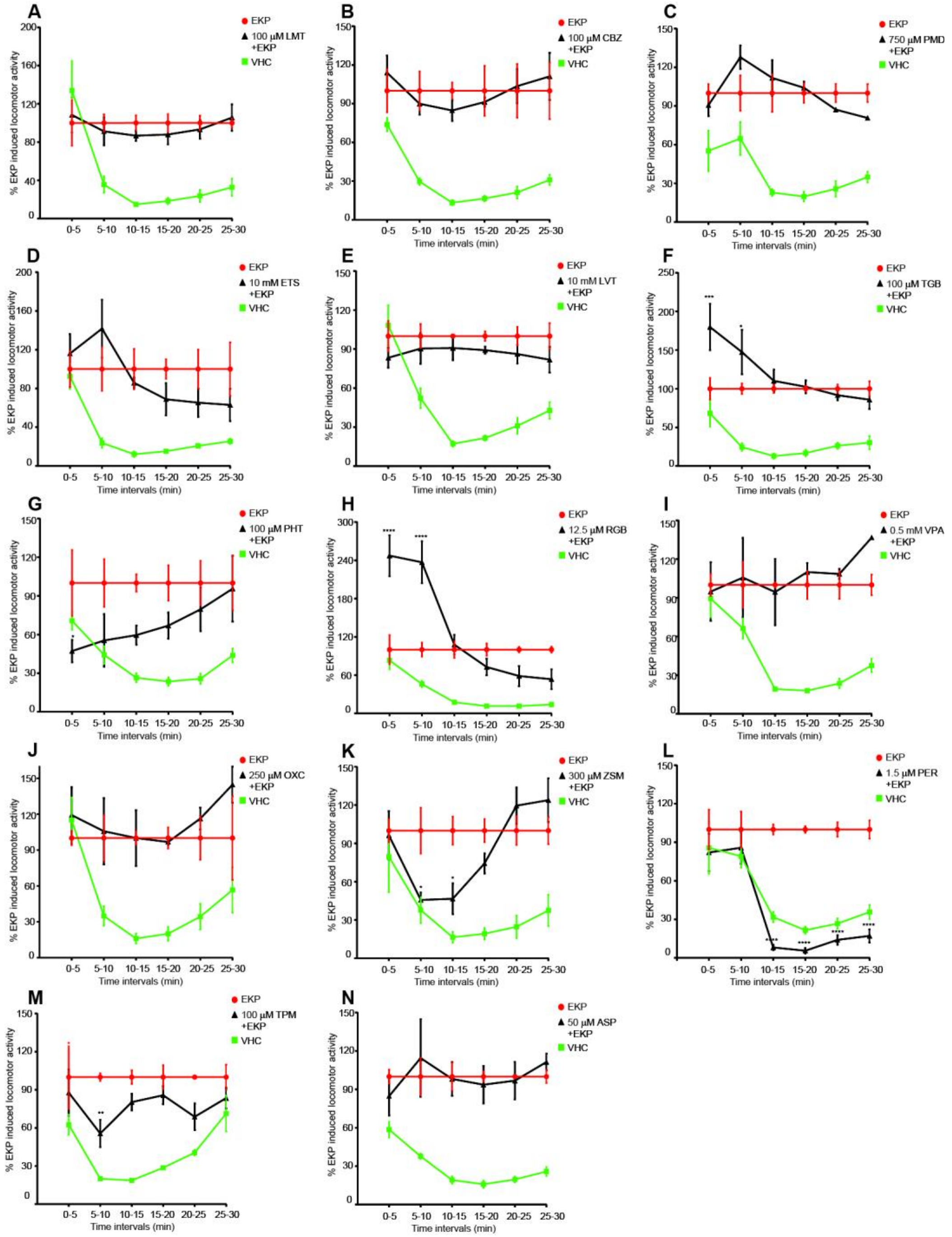

Fig. S2. Detailed locomotor profiles of zebrafish larvae as a response to ASDs treatment in the EKP assay. All results were normalized against EKP controls (set at 100 %). The average larval movement is depicted per 5-min interval (x-axis) of the 30-min tracking session. Time points where the average movement was significantly decreased compared to EKP control (two-way ANOVA) are indicated as*, **, ***, **** (p≤0.05, p≤0.01, p≤0.001 and p≤0.0001 respectively); Error bars represent s.e.m.

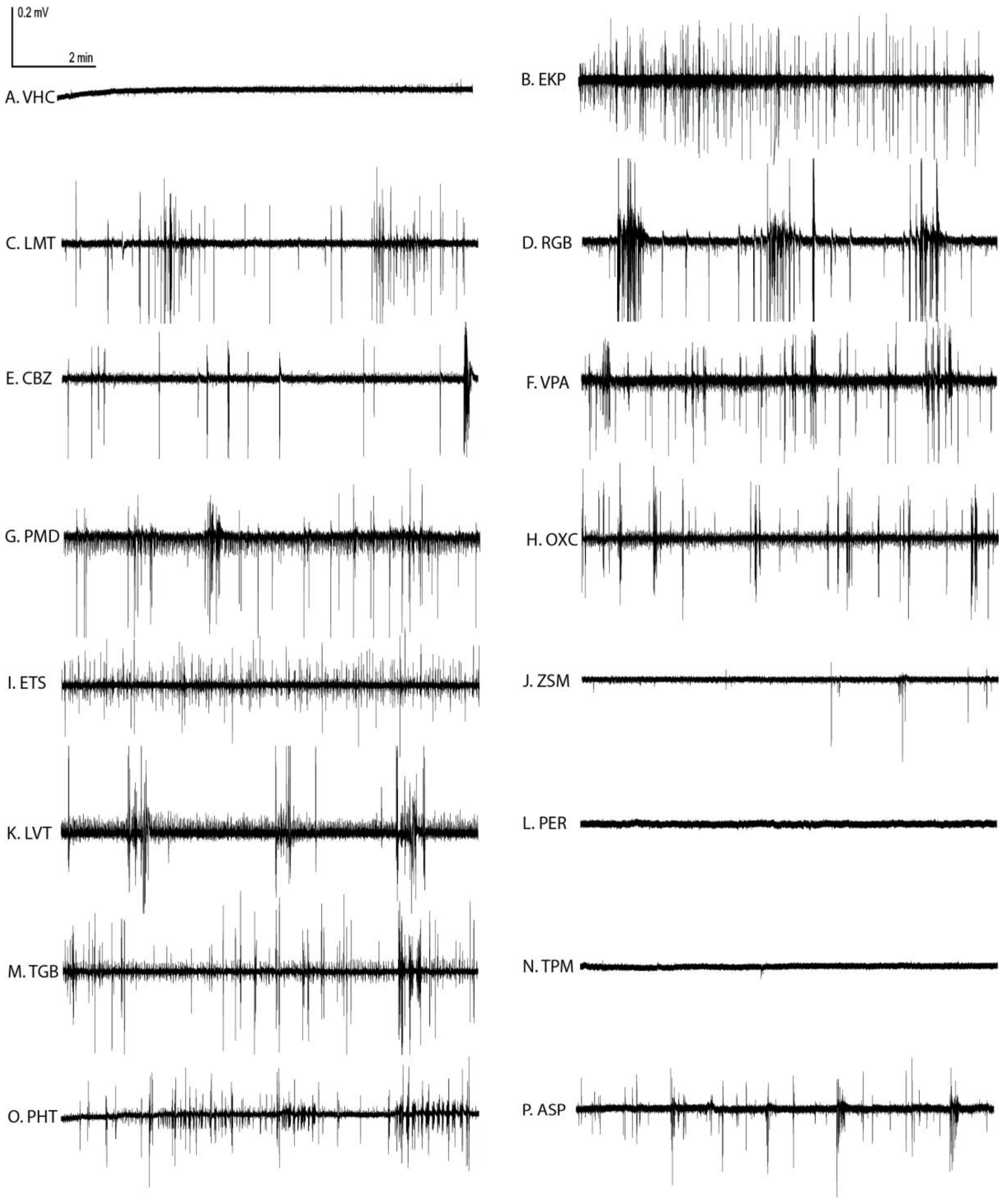

Fig. S3. Effect of ASDs on the electrographic activity in zebrafish optic tecta upon EKP exposure: fragments of representative recording traces. A: VHC (vehicle); B: EKP; (C-P): all other conditions (EKP with pre-treatment of specific ASDs or ASP (aspirin)). Recordings were performed using WinEDR.

Supplementary Tables

β-actin 1	
	Forward CGAGCAGGAGATGGGAACC
	Reverse CAACGGAAACGCTCATTGC
ef1α	
	Forward CTTCTCAGGCTGACTGTGC
	Reverse CCGCTAGCATTACCCTCC
18s	
	Forward TCGCTAGTTGGCATCGTTTATG
	Reverse CGGAGGTTCGAAGACGATCA

Table S1. Sequence of the used primers

	5min	10min	15min	20min	25min	30min
200µM	0,9804	0,5954	0,0769	0,0229	0,0034	0,0019
300µM	0,9944	0,1585	0,0015	0,0002	0,0001	0,0001
400µM	0,738	0,0453	0,0005	0,0001	0,0001	0,0001
500µM	0,5879	0,0001	0,0001	0,0001	0,0221	0,1099
600µM	0,7334	0,0001	0,0001	0,0078	0,1152	0,4566
700µM	0,0394	0,0001	0,0009	0,2186	0,9086	0,9996
800µM	0,0205	0,0001	0,2087	0,9997	0,9976	0,985
	35min	40min	45min	50min	55min	60min
200µM	0,0004	0,0021	0,006	0,0195	0,0065	0,0056
300µM	0,0001	0,0005	0,0024	0,0094	0,0521	0,0898
400µM	0,0086	0,0431	0,1405	0,4734	0,7601	0,8893
500µM	0,2967	0,5935	0,9352	0,971	0,9938	0,9969
600µM	0,9253	0,9995	0,9998	0,9998	0,9999	0,9998
700µM	0,9997	0,9975	0,9965	0,9976	0,9932	0,9957
800µM	0,9516	0,946	0,9527	0,9849	0,9741	0,991
	65min	70min	75min	80min	85min	90min
200µM	0,0088	0,0061	0,0165	0,0218	0,0108	0,0057
300µM	0,0709	0,0959	0,2581	0,6076	0,6291	0,8887
400µM	0,959	0,9994	0,9977	0,9998	0,9998	0,9999
500µM	0,9947	0,9996	0,9997	0,9999	0,9999	0,9997
600µM	0,9999	0,9996	0,9978	0,9971	0,9995	0,9994
700µM	0,9979	0,9933	0,993	0,9877	0,9978	0,9977
800µM	0,9971	0,9905	0,9904	0,9841	0,9976	0,9974
	95min	100min	105min	110min	115min	120min
200µM	0,0321	0,0628	0,0865	0,0738	0,2608	0,285
300µM	0,8395	0,8381	0,9292	0,9896	0,9806	0,9954
400µM	0,9998	0,9999	0,9998	0,9999	0,9999	0,9996
500µM	0,9997	0,9996	0,9999	0,9996	0,9997	0,9978
600µM	0,9979	0,9972	0,9995	0,9994	0,9995	0,997
700µM	0,9976	0,9969	0,9994	0,9994	0,9994	0,9954
800µM	0,9973	0,9949	0,9994	0,9978	0,9994	0,9943

Table S2. Statistical analysis using two-way ANOVA followed by Dunnett's multiple comparison test to compare the EKP- and VHC-treated larval movements of every 5-min interval during 120-min tracking for each of the EKP concentrations used. P-values are shown.

Reference

1. Speare, D.M., Fleming, S.M., Beckett, M.N., Li, J.J. & Bugg, T.D. Synthetic 6-aryl-2-hydroxy-6-ketohexa-2,4-dienoic acid substrates for C-C hydrolase BphD: investigation of a general base catalytic mechanism. *Org Biomol Chem* **2**, 2942-50 (2004).
